# On the Detection and Scope of “Third-Hand Vaping”: A Critical Review of the Literature

**DOI:** 10.3390/ijerph23070940

**Published:** 2026-07-22

**Authors:** Roberto A. Sussman

**Affiliations:** Institute of Nuclear Sciences, National Autonomous University of Mexico, Mexico City 04510, Mexico; sussman@nucleares.unam.mx

**Keywords:** third hand smoke, third hand vaping, adsorption of nicotine, nicotine skin absorption, vaping aerosol residues, dermal exposure

## Abstract

**Highlights:**

“Third-hand smoke” (THS) refers to the persistent toxic residues of aged environmental tobacco smoke (ETS) that accumulate on surfaces and dust in indoor spaces. It is important (and remains an open question) to examine whether an analogous source of exposure has been detected and/or can be generated by environmental vaping aerosol (EVA).

**Public health relevance—How does this work relate to a public health issue?**

- Exposure to persistent aged residues of THS is potentially hazardous for dwellers of indoor spaces, even after smoking no longer takes place.
- Since vaping is a popular alternative to smoking, it is necessary to assess the possible existence and scope of an analogous “third-hand” exposure.

**Public health significance—Why is this work of significance to public health?**

- It is important to fully understand the interdisciplinary connection between health risks and potential hazards to environmental conditions.
- Nicotine is common to smoking and vaping, it can provide a useful connection between their environmental emissions and the scope of their “second-hand” and “third-hand” emissions.

**Public health implications—What are the key implications or messages for practitioners, policy makers and/or researchers in public health?**

- It is important to fully understand the similarities and differences between environmental effects from smoking and vaping when drafting appropriate public policies.
- Understanding the conditions and effects of different exposure routes (inhalation; ingestion; dermal) is essential for all professionals involved in public health and environmental issues.

**Abstract:**

**Background**: “Third-hand” exposure produced by “second-hand” environmental vaping aerosol (EVA) has been reported as a parallel analogue of “third-hand smoke” (THS) produced by residues of environmental tobacco smoke (ETS). **Methods**: To assess the plausibility and scope of “third-hand” vaping exposure, we examine the available evidence on THS phenomenology and the physicochemical properties of EVA. We also evaluate daily intake doses from direct dermal exposure to nicotine under simplified but minimally realistic scenarios for vaping and smoking. **Results**: The only THS phenomenology fully reproduced in EVA is the adsorption of nicotine and its byproducts on indoor surfaces. Unlike significant, measurable THS exposures in normal environments (homes, hospitality venues, cars), third-hand exposures from vaping have only been found under special conditions: laboratory-generated aerosols or in vape shops where excessively abnormal and intense vaping activity takes place. **Conclusions**: The physicochemical properties of EVA are incompatible with an aging process leading to a THS analogue under the environmental conditions in which vaping normally takes place. However, objectively and rigorously evaluating the risks of dermal exposure from smoking and vaping remains an open issue.

## 1. Introduction

Fresh environmental tobacco smoke (ETS) originates from the smoker’s exhalation (mainstream emission (MS)) and the continuously smoldering tip of the lit cigarette (side-stream emission (SS)), with the latter emission comprising most of the mass released into the environment [1,2]. It is also more toxic on a per-gram basis than the exhaled MS smoke [3].

As ETS spreads in indoor spaces, it becomes more diluted but more toxic per gram, since it reacts with common indoor oxidants: ozone, nitrogen oxides, and acids [4]. A set of slow, long-term physicochemical processes mediated by these reactions, known as “aging”, takes place in indoor spaces: nicotine and other volatile and semi-volatile organic compounds (VOCs and SVOCs) in ETS deposit on indoor surfaces and dust particles and can even penetrate directly into human skin and clothes, with further reactions potentially producing new compounds that (as shown in laboratory experiments) might resuspend to form a secondary more toxic aerosol [5].

“Third-hand smoke” (THS) represents the stable residues produced by the aging processes of ETS, i.e., “second-hand smoke”. There are several comprehensive studies and “state-of-the-art” reviews of THS, focusing on its relationship with and evolution from ETS, with evidence from laboratory experiments and field studies, as well as health effects in exposed populations [6,7,8,9,10].

While the period of exposure to fresh ETS is short (hours) and occurs only through inhalation, exposure to THS presents a more complex pattern. Besides inhalation of aged residues that might be resuspended, THS mostly involves exposure via dermal and ingestion routes to nicotine and other VOCs and SVOCs and reaction byproducts adsorbed by (i.e., attached to) dust particles and indoor surfaces: walls, fabrics, objects, clothing, and even in the skin and hair of indoor dwellers [5,11].

THS is an important source of indoor pollution with complex exposure patterns due to the long-term persistence of its residues, which remain long after smoking has ceased, as well as the effects of building structures, ventilation, and varying environmental conditions. Exposure to THS has become a critical public health issue affecting indoor dwellers who unsuspectingly manipulate surfaces, objects, clothing, carpets, or objects inside the cabins of motor vehicles. The exposure of toddlers and children at home is especially concerning, as they are prone to hand-to-mouth motion and crawling on carpets and fabrics [6,7,8].

Importantly, exposure to THS is not confined to adsorption by indoor surfaces; THS exposures also result from partitioning paths from the gas phase to environmental particles, to dust, and even directly to human skin and clothing [12]. Tang et al. [13] examined exposure to TSNAs in realistic THS indoor scenarios along all these partitioning paths, while exposure specifically to ETS and THS in the context of the gas-phase partitioning of nicotine to human skin and clothing was examined comprehensively by Bekö et al. [14].

In the last 15 years, e-cigarettes have become popular consumer products, with an estimated consumer base of over 100 million worldwide in 2023 [15,16]. They generate an aerosol without combustion by heating a liquid mixture (the “e-liquid”) comprising propylene glycol, glycerol, nicotine, and flavor chemicals. Aerosol generation occurs very rapidly and locally along the following steps: (1) thermal energy is supplied to heat a metallic coil in close contact with an organic wick that absorbs a small, localized amount of e-liquid through capillarity; (2) the e-liquid is rapidly heated to its boiling (or near-boiling) temperature to form a vapor; (3) part of the initially supplied thermal energy is absorbed as latent heat for vaporization close to thermal equilibrium at roughly constant boiling temperature; (4) an aerosol with liquid droplets is generated as the user’s inhalation drags, cools, and condenses this vapor.

There is a broad scientific consensus that vaping exposes end users to a significantly lower load of toxic and carcinogenic compounds compared with smoking cigarettes [17]. There is also evidence that their usage contributes to smoking cessation [18]. Nevertheless, vaping remains a controversial issue [19,20]), not only due to its safety profile and long-term effects but also the unintended risks of its use by adolescents and never-smokers.

Just as ETS is the “second-hand” smoke released to the environment, e-cigarettes release a “second-hand” environmental vaping aerosol (EVA). ETS and EVA are environmental pollutants that spread in indoor spaces, whose effect on bystanders is a public health issue that needs to be addressed and continuously monitored. In addition, despite their different properties, nicotine remains the main common compound in ETS and EVA. Since its adsorption and permanence on indoor surfaces is the primary early signal of ETS aging leading to THS, it seems natural to assume that nicotine adsorption in EVA should lead to an analogous aging process and a “third-hand” phenomenology in vaping. Our aim is therefore to probe this hypothesis.

The increasing popularity of vaping implies an increasing presence of EVA, perhaps less so in public indoor spaces than in private spaces and the homes of vapers. Therefore, it is both scientifically valid and in the interest of public health to assess the plausibility, scope, and characteristics of a “third-hand” phenomenology in vaping, produced via EVA aging analogous to ETS aging leading to THS.

However, EVA and ETS are highly distinct aerosols. Therefore, to assess the hypothesis of a “third-hand” phenomenology in vaping, it is necessary to understand their physicochemical properties. The most striking difference between EVA and ETS is the fact that ETS involves a much larger mass release to the environment, since the continuous SS emission comprises most of its mass (60–80% [1,2]), while EVA has no SS emission (no continuous emission from smoldering tip); it therefore represents a truly intermittent and reduced mass release that originates (and lasts) only from the users’ exhalation (MS emission).

Two essential properties of ETS that favor an aging process are the (1) abundance of gas-phase reactive compounds, especially VOCs and SVOCs [1,2,10] and (2) long-term permanence in the environment [1]. Evidence shows that in EVA, nicotine is the only gas-phase reactive SVOC found in significant concentrations [21,22,23], while under normal conditions its rapid evaporation of the droplets and dispersion of the gas phase implies short-term permanence in the environment [21,24,25]. Long-term permanence in the environment occurs in EVA only under special conditions that slow its evaporation and dispersion: confinement of the aerosol in a closed chamber or by continued aerosol replenishment by many users vaping (e.g., in vape shops and at vape festivals) [26].

The published literature reporting the detection of “third-hand” vaping includes three laboratory experiments (Goniewicz and Lee [27], Marcham et al. [28], and Colby et al. [29]) and three observational studies on vape shops (Khachatoorian et al. [30,31] and Son et al. [32]). The three observational studies and two of the experiments [27,28] measured substantial levels of surface densities of adsorbed nicotine and its byproducts, including TSNAs.

However, these measurements were accomplished under very special conditions that do not correspond to the environmental conditions (inhalation and dermal exposure) in which vaping usually takes place. In two experiments [27,28], aerosol was injected via syringe into a closed chamber, leaving it confined to the chamber for long periods. The other experiment [29] followed an even more artificial protocol, taking 5 days to force a phase partition on an injected aerosol in a small chamber. While there is mention of a third-hand exposure from vaping aerosols in [33], the article only cites [27,28] and speculates on this possibility.

The three observational studies measured significantly high surface densities of nicotine and TSNAs during long periods (up to 6 months) on fixed and implanted surfaces inside vape shops. However, the highest surface densities were measured on highly adsorptive external fabrics that the researchers brought and placed inside the vape shops. Those measurements are evidently artificial adsorption sinks and are unrepresentative of nicotine adsorption in these vape shops.

However, vape shops in general have never been representative of the (indoor and outdoor) environments where vaping activity normally takes place; they are small businesses with modest furnishings that many vapers sporadically visit for short periods to buy supplies. Since vaping was allowed in the vape shops sampled in these studies during 2018–2020, high nicotine surface densities were measured in correlation with abnormally high vaping activity.

Measuring significant surface densities of adsorbed nicotine requires abnormal conditions, which stands in sharp contrast to field studies of THS, in which measurable surface densities have been obtained under normal conditions in private homes, hotels, hospitality venues, motor vehicles, and homes housing medically fragile children [34,35,36].

Comparing field studies shows that a measurable third-hand phenomenon such as nicotine adsorption occurs more naturally and more efficiently with ETS, while to be significantly measurable, it must be forced in EVA. In all instances (lab experiments and vape shops), nicotine adsorption was significant because evaporation and dispersion of EVA were prevented via confinement in a laboratory chamber or through excessive aerosol replenishment in a busy vape shop.

Finally, it is important to stress that high levels of adsorbed nicotine and TSNAs measured on indoor surfaces do not determine dermal exposure risks. Hence, we evaluate the actual dose intake of nicotine and its byproducts under simplified but minimally realistic scenarios of direct dermal exposure to adsorption sinks in indoor surfaces.

The section-by-section plan of this review is described as follows. As necessary background material to assess the plausibility and scope of a “third-hand” exposure from vaping, Section 2 provides a summary of experimental evidence and field studies on THS, while Section 3 presents a summary of the physicochemical properties of EVA. Section 4 presents and describes published studies (experimental and observational) that have found effects that can be associated with a “third-hand” phenomenology in vaping aerosols. The results of these studies are analyzed and discussed in detail in Section 5. In Section 6, we present two scenarios based on a risk model on dermal exposure to adsorbed nicotine in indoor surfaces, while also commenting on two studies that examined cancer risks from dermal exposure to TSNAs (the parameters of the risk model are described in the Appendix). Our conclusions are stated in Section 7.

## 2. Background: Third-Hand Smoke (THS)

As aerosols spread indoors, their particulate and gas phases sustain kinetic interactions with surfaces mediated by (mostly) oxidation reactions with prevalent pollutants (whether generated indoors or outdoors). If their gas phase contains volatile and semi-volatile organic compounds (VOCs and SVOCs), aerosol–surface phase partitioning drives the adsorption of these gases, forming thin liquid films on the surfaces. We identify the gases as ”adsorbates” and the surfaces as ”adsorbents”. The seminal work on equilibrium-phase partitioning of VOCs and SVOCs was published by Weschler and Nazaroff [12].

As adsorbed VOCs and SVOCs become long-term stable surface ”reservoirs”, some of the material is desorbed and re-suspended, undergoing further interaction with environmental pollutants to produce new gaseous compounds and particles through nucleation. The evolution of these processes in a primary organic aerosol (POA) is known as its “aging”, which might involve sufficient new gaseous and particulate byproducts to define the aged aerosol as a new ”secondary organic aerosol” (SOA) distinct from the POA.

THS is the product of these processes acting on ETS as a specific POA. However, ETS is not the only aerosol subjected to this phenomenology, since equilibrium-phase partitioning also occurs with other indoor aerosols as adsorbates whose gas phases contain VOCs and SVOCs. See a recent review on various organic primary and secondary aerosols distinct from ETS [37,38]. Other examples are aerosols produced by cooking [39], cleaning liquids [40], sprays [41], and candles [38].

The aging of ETS leading to THS in indoor spaces and the full phenomenology of THS is extensively described in the reviews [6,7,8,10], while a full summary of the literature up to 2018 can be found in [9].

### 2.1. Experimental Evidence

Freshly emitted ETS is a mixture of mainstream (MS) and side-stream (SS) emissions, with the latter comprising most of its mass [1,2]. As it spreads indoors, it interacts with pollutants and dilutes via evaporation of volatile compounds. The large share of non-volatile compounds in its particulate phase keeps it airborne for long periods.

As equilibrium-phase partitioning sets in, gaseous VOCs and SVOCs (particularly nicotine) deposit on dust particles and on indoor surfaces as adsorbents (including furniture, clothing, and even inside the human body). Since these compounds remain adsorbed for long periods (even months and years without anyone smoking), dermal and ingestion exposures become possible [6,7,8]. An updated comprehensive analysis of various aspects of THS phenomenology and exposure risks to TSNAs is found in [13].

Although nicotine adsorption on indoor surfaces also happens with fresh ETS, it is a clear signal (at least the first stage) of THS formation. Either in gaseous form or adsorbed in dust and surfaces, nicotine sustains oxidation reactions with nitrogen acids and oxides and ozone [4,11] to form many byproducts, including tobacco-specific nitrosamines TNSAs, specifically 1-(N-methyl-N-nitrosamino)-1-(3-pyridinyl)-4-butanal (NNA) and 4-(methylnitrosamino)-1-(3-pyridyl)-1-butanone (NNK).

The role of nicotine as a tracer of THS is further illustrated by the fact that one of its byproducts, NNA, is absent in fresh ETS (as opposed to NNK) but is detected when analyzing oxidizing reactions on nicotine in ETS, indicating that it is a secondary product of aging, not a compound emitted in ETS [5]. Nicotine is the most abundant SVOC in the gas phase of ETS and is also a highly efficient adsorbate, as has been proved empirically in various experiments (see (Singer et al., 2004) [42] below). In what follows, we summarize various key laboratory studies on THS phenomenology:

- Singer et al. [42] specifically examined, under the same experimental conditions as their previous 2002 paper but also considering a fully furnished room, adsorption/desorption rates for 20 VOCs and SVOCs common to indoor spaces, again showing nicotine to be the most efficient adsorbate. Full furnishing was shown to produce a more complex pattern of adsorption sinks.
- Singer et al. [43] examined 26 gas-phase VOCs and SVOCs identified as ETS markers in three ventilation regimes in a 50 m^3^ model room furnished with typical household material. Non-adsorptive compounds were found to remain stable under varying ventilation conditions, while nicotine and 3-ethenylpyridine remained adsorbed 3 days after smoke injection.
- Schick et al. [44] verified that nicotine, cotinine, and TSNAs are not the only compounds adsorbed, since most polycyclic aromatic hydrocarbons (PAHs) released during smoking in homes and public places also deposit on room surfaces and fabrics. However, the main challenge with finding airborne compounds that can serve as tracers of THS is the fact that prospective THS compounds also appear in ETS. Most evidence on THS phenomenology beyond nicotine adsorption and the formation of its byproduct follows from controlled laboratory experiments and simulations.
- Sleiman et al. [7] detected 58 VOC and SVOC compounds in ETS: (a) nitrogenated (amines and nitriles), (b) aromatic hydrocarbons, (c) carbonyls and chlorinated, and (d) alkanes and alkenes. Since all are present in ETS, they focused on those whose concentration increased from 2 to 18 h, identifying acetonitrile, 2,5-dimethyl furan, and 2-methyl furan as possible markers of the transition from ETS to THS. However, those findings have not been verified in field studies outside the authors’ experiment.
- Richardot et al. [45] examined prospective THS compounds through a non-targeted analysis of settled house dust samples from smokers’ and non-smokers’ homes. Among the 42 selected compounds, 26 were statistically more abundant (*p* < 0.10) in dust from homes of smokers; seven were tobacco-specific compounds, two of which (nornicotyrine, 3-ethenylpyridine) had not been reported in house dust before.
- DeCarlo et al., 2018 [46] identified a reduced nitrogen component predominantly found in the indoor environment, contributing 29% of the indoor submicron aerosol mass, providing a good indication of THS compounds partitioning from interior surfaces to the gas phase and then aerosol phase. In a laboratory study, Petrick et al. [47] modeled SOA formation from nicotine-ozone-NOx reactions.

### 2.2. Field Studies

Beyond the laboratory, practically all field work on THS comprises evaluating the presence of nicotine and byproducts (including TSNAs) on dust and surfaces in a wide variety of indoor spaces, e.g., homes, cars, and hotels. The method of evaluating nicotine’s presence involves surface wipe sampling, with wipes being standardized surfaces that act as calibrated environmental markers—see [48] for details. The following key studies provide field evidence of nicotine adsorption in many indoor spaces and venues:

- Matt et al. [34,35] and Quintana et al. [48] sampled surface densities of nicotine (in µg/m^2^) from surface wiping to distinguish smoking and non-smoking spaces. In [48], surface densities were sampled in a wide variety of indoor environments, suggesting a preferred cut-off value of 10 µg/m^2^ that clearly identifies non-smoking environments but included a minority of indoor spaces with low indirect exposure to ETS.
- Mat et al. [34] measured nicotine surface densities in cars owned by smokers that do not smoke aboard (5.09 µg/m^2^) and those who do (8.61 µg/m^2^), while in non-smokers’ cars with a smoking ban the measurement was 0.06 µg/m^2^. A proposed cut-off of 0.14 µg/m^2^ separated all non-smoking cars and 82% of smokers’ cars in which there was no smoking.
- Matt et al. [35] showed that non-smokers buying houses whose previous owners smoked had six-times more nicotine residues in their living room than houses bought from non-smokers: 10.04 µg/m^2^ vs 1.52 µg/m^2^.
- Northrup et al. [36] examined adsorbed nicotine using surface density wipes in the homes of vulnerable children with smoking and non-smoking parents. No nicotine density was measured in non-smoking homes, while in smoking homes with smoking restricted to the outdoors, the density decreased to 1.7 µg/m^2^ (it reached 19.1 µg/m^2^ with three smokers without an indoor ban).

## 3. Properties of the Environmental Vaping Aerosol (EVA)

Since nicotine is the most common and abundant SVOC in EVA and ETS, and as nicotine adsorption and its byproducts play such an important role in defining THS, it would seem natural to assume that something analogous to THS should occur in vaping. However, this reasoning is misleading, since EVA and ETS are chemically and physically very different. In fact, that nicotine is an important constituent in both aerosols could be the only significant similarity between them.

The following characteristic properties of EVA clearly show that it does not favor the phenomenology associated with an aging process that defines THS. As we discuss below, nicotine adsorption from EVA can be substantially different from adsorption from ETS.

### 3.1. There Is No Side-Stream SS Emission

This fact has important consequences: nicotine is exclusively released to the environment via user exhalation, and users retain on average more than 90% of inhaled nicotine. This was established by St. Helen et al. [49] by sampling exhaled breath of users of vaping devices available up to 2016, but Hua et al. [50] found near 100% retention in users puffing newer Juul devices. Vaping therefore releases a significantly lower nicotine mass for adsorption in indoor surfaces. While smokers also retain most of the inhaled nicotine, a large nicotine mass is continuously released through the SS emission.

In addition, users retain most of the inhaled compounds besides nicotine: > 80% glycerol, > 90% propylene glycol [48,49,50,51] and close to 100% of aldehydes and VOCs [51,52,53]. Consequently, once exhaled into ambient air (a large-volume thermal reservoir), the aerosol is more diluted than the inhaled one, and its chemical content is simpler than that of the inhaled aerosol.

Retention of most compounds has been examined by Asgharian et al. [54] in their modeling of physical phenomena that affect the inhaled aerosol in its passage through respiratory tracts characterized by 37 °C and 95–100% relative humidity. In particular, the main involved processes are hygroscopic growth of droplets, impactation and gravitational settling, and diffusion of ultra-fine droplets. The predictions of this modeling fully agree with the results of sampling exhaled breath.

In contrast, a much larger nicotine mass to be adsorbed should be released to the environment with ETS from smoker exhalation (MS emission) and the continuous SS emission that constitutes 60–80% of ETS [1,2].

### 3.2. Protonated vs. Non-Protonated Nicotine

All existing experiments summarized on THS involved predominantly non-protonated (free-base) nicotine in the gas phase, which is much more volatile than its protonated form, which is only delivered through the particles [55]. These properties influence free-base nicotine in the equilibrium gas-phase partitioning associated with adsorption into surfaces, especially household furniture, carpets, wood, and upholstery.

Practically all vaping activity before 2018 relied on e-liquids with overwhelmingly predominant free-base nicotine [55], the same type of nicotine as in the THS studies. Although free-base nicotine can also be found in the droplets (particulate phase), it evaporates into the gas phase almost immediately after exhalation [56]. Before 2018, many vapers puffed high-powered “mod” devices that released larger aerosol masses [57], including a larger mass of nicotine (even though these devices utilize e-liquids with low nicotine concentrations).

In tobacco smoke and e-cigarette aerosol, the degree of protonization can be expressed in terms of the ratio α of unprotonated free-base nicotine to protonated nicotine. This ratio determines inhalation harshness, being higher for more unprotonated nicotine with a higher free-base ratio (α ~ 1) and lower for a more protonated proportion (α ~ 0). Therefore, a more protonated nicotine is ideal for delivering high concentrations even through low-powered devices and with little harshness for users (see [55]). These properties of nicotine likely contributed to a market incentive to rely on a more protonated nicotine, which requires increasing its PH to make it more acidic. This transformation led to near-fully protonated nicotine salts formed with benzoic acid.

Before 2019, the vaping market was dominated by first- and second-generation devices and tank models (Jiang et al., 2023), whose e-liquids had nicotine with almost 100% free-base nicotine (α ~ 1) (see [57]) and mostly in lower concentrations (<10 mg/mL). Since 2020, the vaping market shifted to higher nicotine concentrations and low-powered devices, at least in part because this shift is favored by more protonated nicotine.

As early as in 2021, disposables with protonated (salt-based) nicotine became the most frequently used devices [58]; the authors of [59] reported the use of disposables among 38% of 15–20-year-olds and 32% of people aged 21 and older. This tendency has increased among youth, with the 2024 New Youth Tobacco Survey [60] reporting an even larger share of devices based on protonated nicotine: 55.6% disposables and 15.6% prefilled or refillable pods or cartridges, with tanks constituting only 7%. Similar developments are reported in the UK: 60% of vapers use disposables and pod-based devices, with the remaining 40% using refillable devices [61].

This change in the vaping market works against the efficiency of nicotine adsorption because nearly protonated nicotine is much less volatile and is contained not in the gas phase but in the droplets and barely evaporates [56]. This seems to be the worst-case scenario for nicotine adsorption and for THS following equilibrium-phase partitioning: an EVA with a low mass content of non-volatile nicotine delivered only through aerosol droplets.

### 3.3. EVA Has a Simpler Chemistry than ETS

The complexity of the chemistry of ETS is well-known [1,2], with more than 4000 compounds having been detected. Most toxic and carcinogenic compounds in the MS and SS emissions are present in diluted concentrations in ETS compared with MS, plus extra compounds from reactions with environmental pollutants. In contrast, fewer than 150 compounds have been detected in EVA (around 70 compounds are listed in [22]), with four (PG, VG, nicotine, and water) representing almost the total aerosol mass [23].

The chemical complexity in vaping aerosols is predominantly in the organic byproducts (especially aldehydes) produced by thermal processes during the formation of the aerosol that users inhale. These byproducts form as part of the aerosol generation, either by users or puffing machines activating a vaping device. The e-liquid in the wick is vaporized at the boiling temperature of the mixture (188–288 °C), with liquid droplets formed by the subsequent cooling and condensation as the vapor is drawn out (forced convection) by the user’s inhalation. This heating process produces organic byproducts through thermal degradation (low energetic pyrolysis) reactions of the solvents: propylene glycol and glycerol and of the flavor chemicals [23]. While propylene glycol can react with ozone [62], it is not a highly reactive compound, and even less so in the small concentrations found in the rapid dispersing gas phase of EVA.

The most abundant byproducts, aldehydes, are overwhelmingly retained by users [48,49,50,51,52,53] and no new compounds are generated under the conditions inside the respiratory system, since the thermal degradation reactions only activate significantly at temperatures of around 200 °C [23]. Consequently, the exhaled aerosol is even chemically simpler than the inhaled aerosol, which is already vastly chemically simpler than the MS of tobacco smoke.

One of the most detailed studies on the chemical composition of EVA in a vaping atmosphere (as opposed to chamber studies) was conducted by van Drooge et al. [22], who showed that after 12 h of ad libitum vaping in five daily sessions, the particulate and gas phases of EVA are practically indistinguishable from background environmental levels without vaping. The trace remnant of the particulate phase after 12-h vaping sessions in 5 days comprised residual glycerol and nicotine, while the only measurable sign of the gas phase was a slight increase in formaldehyde below safety standards and comparable to non-smoking environments. Similar results for the gas phase were obtained in [21].

Even under the abnormal conditions of vape festivals, where hundreds of vapers congregate, the resulting gas-phase atmosphere comprises almost only the solvents propylene glycol and glycerol [26]. Of the 26 tracers of ETS displayed in the figures in [42], only nicotine is significantly found in EVA; the rest are either not detected or, like acrolein, are detected in minute quantities that are almost indistinguishable from non-vaping background levels [23].

### 3.4. EVA Remains in the Environment for Short Times

EVA evolves in respiratory tracts at 37 °C and 95% RH, since users retain >80% of glycerol and >90% of propylene glycol and nicotine; as it is exhaled, it flows into (in general) colder and drier environmental air [54]. This passage from a saturated medium to a drier one strongly favors the rapid evaporation of very volatile glycol-based aerosols [63] and free-base nicotine in liquid droplets, with diminished droplets made of glycerol (and nicotine if protonated). The rapid evaporation of droplets [56] (half-life of <20 s per puff measured in laboratory puff sequences) and low molecular mass of gaseous compounds rapidly dilutes and disperses EVA to achieve thermal equilibrium with environmental air as a thermal reservoir [24,25].

Only under abnormal conditions of intense vaping activity—vape shops or vape festivals—do the evaporation, dispersion, and dilution of EVA slow due to continuous aerosol injection, leaving residual levels of aerosol for hours [26]. However, because evaporation occurs at ambient temperatures and pressure, the slower rate does not modify the aerosol’s chemical composition or trigger reactions between the solvents and environmental pollutants that form byproducts.

In contrast, ETS is a complex mixture of two combustion originated aerosols (the MS and SS emissions) and environmental pollutants. In its gas and particulate phases, ETS contains many reactive compounds with a high share of non-volatile content, as well as non-volatile, ultra-fine particles in the particulate phase, all of which persist in the environment for a long period. Its half-life (also measured in the laboratory) is 30–60 min per puff [24,25].

## 4. Evidence of a “Third Hand” Vaping Aerosol

There are published studies claiming that a third-hand phenomenology analogous to THS is not only plausible, but fully demonstrable, and these studies can be classified as follows:

- Three laboratory experiments [27,28,29] Primary vaping aerosol is artificially generated with syringes and injected into a chamber. In two experiments [27,28], the target was to measure the surface density of adsorbed nicotine in household fabrics and materials inside the chamber, leaving these samples exposed to the aerosol for prolonged time. One experiment [29] mixed the aerosol with another aerosol and left them interacting for 5 days, producing an incipient new aerosol.
- Three studies on nicotine adsorption from EVA in vape shops [30,31,32]. Researchers measured the area density of nicotine and its byproducts on fixed surfaces and on fabrics they brought to place on the surfaces. The area density on the fixed surfaces and the fabrics was measured for extended periods (up to 3 months).

In all studies, adsorption and desorption of nicotine and its byproducts are reported. The authors describe this adsorption as evidence that “third-hand vaping” has been discovered.

### 4.1. Laboratory Studies

- Goniewicz and Lee [27]: The experiment delivered 100 puffs in 1.5 h from three brands of e-cigarettes, injected directly into an exposure chamber by attaching the e-cigarettes to a 100 mL syringe via rubber connector. Surface wipe samples were taken from five indoor 100 cm^2^ surfaces (window, walls, floor, wood, and metal). Significant increases in the amount of nicotine were measured on surfaces, with the largest occurring on the floor and glass windows. The average amount of nicotine was 205 µg/m^2^ (range 0 to 550 µg/m^2^).
- Marcham et al. [28] placed glass and cotton samples on a Petri dish in a laboratory chamber of volume 1 m^3^. Aerosol was injected via 49 puffs in 15 min through a 500 mL syringe connected to a sub-ohm vaping device. The exposed samples were left for 45 min and then extracted for analysis. The mean amounts of adsorbed nicotine were 0.75 µg/cm^2^ (cotton) and 0.125 µg/cm^2^ (glass). Statistical modeling predicted surface concentrations to reach background levels after 4 and 16 days for glass and terrycloth, respectively.
- Colby et al. [29] demonstrated how to obtain new “particles” re-emitted (“revolatilized”) from specially prepared “residues” of vaping aerosols (from a Juul device). The “residues” require 5 days of intricate chemical procedures, diligently applied in a small, closed chamber. This is a summary of their protocol: the experiments started with puffing a Juul (puff duration: 3–4 s) with a sterile 60 mL syringe to inject the aerosol into a stainless-steel cylindrical chamber with 37.1 L volume. The aerosol was then mixed and flushed with air to retain only the residues left undisturbed until the next day, when the chamber was sampled and analyzed. After filtering laboratory air, laboratory-generated ammonium sulfate (AS) seed aerosol was introduced into the chamber. This experimental procedure was performed for 1, 2, and 5 days. The aerosol was finally sampled.

This study shows how much the aerosol must be chemically coerced to obtain a “third-hand” product (a new artificial aerosol from re-emitted particles). A syringe generated aerosol emitted in four puffs from a Juul aerosol would evaporate in 10–20 s if released into a natural air environment (not injection into a chamber). This has been measured experimentally in aerosols generated by a syringe [63] for volatile glycols. It would be impossible to conduct the experiment of Colby et al. [29] outside their laboratory.

The chemical procedures are correct and the experiment is technically interesting because, contrary to previous studies, it did not rely on nicotine adsorption, since the Juul aerosol contains non-volatile protonated (salt-based) nicotine and (most likely) does not adsorb. While Colby et al. recognize that their experiment is very idealized, they still mention the “potential” for transport of residues to the environment (a “third-hand” effect) and comment on studies conducted inside vape shops.

The merit of these experiments is in finding special laboratory conditions to facilitate measuring significant levels of nicotine adsorption from vaping. However, the experiments are unrealistic: no vaper vapes compulsively into samples, less so inside a chamber. The aerosol injection in these experiments is also excessive (100 puffs in 1.5 h and 49 in 15 min). This excess puffing, together with the need to lock the aerosol in a chamber, is necessary to slow the evaporation and force as much nicotine as possible to deposit on the samples. More normal conditions would probably yield negligible adsorption.

### 4.2. Studies on Vape Shops

A study by Son et al. [32] examined environmental variables (CO_2_, NO_2_, PM_2.5_ aldehydes, and air nicotine) in five vape shops. They also measured the surface density of adsorbed nicotine and detection of NNA and NNK byproducts. The study examined the environmental variables correctly, but the authors reached questionable conclusions on the issue of “third-hand vaping”, to which they dedicate a full subsection; see Discussion in Section 5 for further comments. Their protocol was as follows:

Samples of surface nicotine and TSNAs were collected using wipes on fixed surfaces in each vape shop: a showcase, a TV set, a picture frame, and the floor. However, the authors brought their own surfaces (the “materials”) on which to measure adsorbed nicotine and TSNAs. These were external materials bought from local retailers and brought to the vape shops by the researchers. They were pieces of glass (5 in × 8 in), cellulose filter paper (5 in × 8 in), baby clothing material (5 in × 8 in, 100% Cotton made), rubber ball (5 cm in diameter), and a fur ball (polyester) purchased from a baby toy retailer.

Notice that only the fixed surfaces are typical of a vape shop. The samples brought by the authors are external implants not willingly (or even accidentally) placed by the owners or consumers of the vape shops. These samples would not be in the vape shops without the authors’ intervention.

Table 1 (below) shows the surface density of adsorbed nicotine, NNA, and NNK in the natural and implanted surfaces. As expected by the authors, the surface densities of nicotine, NNA, and NNK were substantially higher in the implanted surfaces than in the natural ones.

It is evident that the only real legitimate values obtained for nicotine adsorption and TSNA presence are those collected from the fixed surfaces, which are much lower than those gathered from the authors’ implants. Placing highly adsorptive external samples artificially enhances nicotine sorption and TSNA levels. Vape shops are adult venues; therefore, it does not seem reasonable (or useful) to place children’s garments in them.

Based on the high surface densities of nicotine and TSNAs detected, Son et al. declared that they had found a “THA” (third-hand aerosol) comparable and analogous to THS. They remarked that the nicotine and TSNA levels were even above those reported in THS field studies. However, as we show in the discussion of Section 5, their interpretation of these measurements is questionable.

The studies by Khachatoorian et al. [30,31] were also conducted in vape shops, with the first study containing most of the useful data. The authors defined adsorbed nicotine and its byproducts, including TSNAs, as “E-cigarette aerosol residue” (ECLEAR). As in Son et al., the two studies by Khachatoorian et al. introduced externally implanted fabrics. They also sampled “ECLEAR” in the living room at the home of a vaper, but they only conducted measurements on an external cotton fabric brought by the researchers and not on the intrinsic surfaces of the living room.

The authors placed commonly used household materials (cotton, polyester, or terrycloth towel) inside (1) the living room of a vaper for 6 months and (2) in a vape shop. The materials were collected at the following stages: 6, 7, 18, 24, and 48 h, 1 week, and 1 month. Evidently, as mentioned before, the household materials brought by the authors would be out of place in a vape shop. Interestingly, the terrycloth towel (a highly hygroscopic fabric) produced the largest yields. The units used by the authors, ng/g, can be converted to ng/cm^2^ by multiplying ng/g values by the towel area density g/26.125 cm^2^ in the vape shop and g/44.16 cm^2^ in the vaper’s living room. These were their results:

- In the vaper’s living room, the authors mention having placed “*six sets of cotton and polyester fabrics hung across the bottom shelf of a bookcase above the computer*”. The mass per area was 1 g for 43.18 cm^2^ (cotton) and 41.91 cm^2^ (polyester). The surface density had small variations over 6 months, ranging between 2000 and 5000 ng/g, occasionally reaching 5100 ng/g. These values are equivalent to the range 0.45 to 1.15 mg/m^2^. Cotinine and other byproducts were barely detectable. No TSNAs were detected.
- At the rear of the shop, the nicotine density increased between 6 h and 1 month from 24 to 679 ng/cm^2^. Lounge area readings were 19–786 ng/cm^2^. The largest density was in the display case, increasing from 32 to 10,914 ng/cm^2^. Cotinine varied from 7 to 8 ng/cm^2^, while NNA reached 3 ng/cm^2^ in 1 month in the display case.

The surface densities of all byproducts of nicotine in the vaper’s living room were relatively small and negligible, only reaching high levels (1.15 mg/m^2^) in the highly hygroscopic external towel placed by the researchers. In contrast, the nicotine surface concentration detected in the terrycloth towel on the display case in the vape shop is truly enormous at 108 mg/m^2^, larger than any adsorbed nicotine surface density registered in THS research. However, this is an artificial and unrealistic scenario, since a terrycloth towel is normally found in bathrooms and gyms and is unlikely to be a natural feature inside a vape shop.

Melstrom et al. [64] sampled nicotine adsorption from aerosols emitted by three vapers vaping ad libitum for 2 h in a small room measuring 57 m^3^, resulting in a surface density of 6.9 µg/m^2^, a value much lower than those obtained by the studies on vape shops. In a follow-up study [65] published by the same authors, which was conducted in the same room and involving three vapers puffing ad libitum, 983 puffs were registered in two hours. It is therefore very likely that the 6.9 µg/m^2^ surface density resulted from a very intense 2-h-long puffing activity in a small room (a scenario unrepresentative of normal routine vaping, since vapers do not normally puff hundreds of times in 2 h).

## 5. Discussion: Vape Shops

Some comments are necessary to provide appropriate context for the studies by Son et al. and Khachaturian et al. on vape shops. The studies were published in 2019–2020, but the field work was likely conducted a year before their acceptance and publication (the supplementary file of the first study by Khachaturian et al. [30] describes field observations performed in 2014–2015). Many US-based vape shops at the time (2014–2019) also acted as social hubs, where smokers explored vaping options and current vapers congregated to socialize for short time periods while buying supplies. Vaping was allowed in the premises that were indoor spaces with relatively small capacities (typically < 300 m^3^), in which an abnormally intense vaping activity took place.

Another important feature of the vape shops sampled by Son et al. and Khachetoorian et al. is their modest furnishing. As in [42], the adsorption of nicotine and other SVOCs differs (say) between extremes: empty chambers and fully furnished rooms or indoor spaces. In an empty experimental chamber with an adsorptive sample (fabric or carpet) inside, there are two adsorption sinks that become large surface reservoirs: the walls and the sample. However, nicotine adsorption becomes distributed among the many sinks and reservoirs that exist in a fully furnished room. The modest furnishing of vape shops puts them between these two extremes but closer to the environment of the empty chamber. This likely (at least) partly explains the high nicotine and byproduct surface densities measured in these studies.

However, it is evident that vape shops, either those examined in these studies in 2018–2019 or vape shops today, represent a very special scenario distinct from the normal vaping routine experienced by millions of vapers worldwide. Vapers do not visit vape shops daily or frequently, only sporadically, and do not stay for hours at a time. While the authors mention this special condition, they do not seem to be aware that their measurements were taken under abnormal and infrequent conditions and are thus unrepresentative of mainstream vaping.

### 5.1. Implanted Samples

Son et al. and Khachatoorian et al. brought samples (towels, baby clothes, paper, glass) and placed them inside the vape shops to measure nicotine and TSNA surface densities. The samples remained for long periods (Son et al.: 14 days; Khachatoorian et al.: up to 1–6 months). However, surface densities measured on this externally implanted material do not represent a real quantitative evaluation of nicotine adsorption in vape shops: they are simply artificial adsorption sinks placed inside the vape shops. The only realistic evaluation in these two studies follows from measurements using wipes on fixed surfaces inherent to the vape shop and not introduced by the researchers for an experiment.

It is hard to find a reason justifying these implanted samples, since under the conditions prevailing in many vape shops in 2018–2019, it was expected that substantial yields of adsorbed nicotine and byproducts would be measured on wipes placed on their natural fixed internal surfaces. In fact, Son et al. and Khachatoorian et al. measured non-negligible significant surface densities in the natural inherent surfaces. Notice that these external samples are highly efficient adsorbent sinks (towels, cellulose paper, cotton clothing); hence, it is not surprising (interesting, but very artificial) that nicotine and TSNA densities were much higher than in the natural samples (see the comparison in Table 1).

Son et al. explicitly mention placing this external material in the spot with more intense vaping activity: “*The five materials were left in each vape-shop for 14 days on a shelf near a main countertop where e-cigarette users were frequently seen vaping*”. The only valid justification for this decision is the authors’ attempt to explore the possible adsorption effects under the most extreme vaping regime, which is theoretically interesting but irrelevant to assess the safety profile of vape shops.

Khachatoorian et al. placed terrycloth towels inside the vape shop: “*on the display case towards the front of the shop, on the lounge table between the two couches, and hung on the chalkboard towards the back of the store*”. After one month, the towel placed on the display case toward the front of the vape shop had a nicotine surface density of 108 mg/m^2^, an enormous value larger than any surface density measured in the field studies of THS.

A terrycloth towel is not expected to be hung inside a vape shop where customers move about. Vape shop owners would naturally place this type of towel in their bathroom, not in the display case that is the most active spot in their shop. Since this type of towel is very hygroscopic (efficiently absorbs water) and base-free nicotine is very volatile, it is an extremely efficient adsorption sink that, after a month, will evidently capture an enormous amount of nicotine and water vapor. This is a very artificial measurement, akin to assessing the density of fish in a pond by placing tons of bait to attract hundreds of fish and then reporting a very high fish density.

### 5.2. Nicotine Surface Density in a Vaper’s Home

Khachatoorian et al. sampled nicotine and byproducts densities in six fabrics made of cotton and polyester in the living room of a vaper. After leaving these fabrics for 6 months, during which deposited aerosols accumulated, the nicotine surface density increased significantly (from 0.45 to 1.15 mg/m^2^ in one towel), but cotinine and other byproducts were barely detectable, and TSNAs were not detected.

Although surface densities in the vaper’s living room were much lower than densities in the vape shop (by a factor of 1/60 of the maximal density of 108 mg/m^2^), these densities are still artificial overestimations because they were evaluated on implanted samples instead of the inherent surfaces of the vaper’s living room. It is not impossible to place a towel in a living room, but it would be extremely odd to keep it there (in a *bookcase above the computer)* for 6 months, gathering nicotine. In real life, this towel would be machine-washed, sent to the laundry, or placed in a bathroom closet.

### 5.3. Comparison with Nicotine Adsorption in Smokers’ Homes

Son et al. compared measured surface densities with levels in smokers’ homes

- *“However, thirdhand exposure to nicotine and carcinogenic TSNAs could be much higher in vape-shops than that caused by cigarette smoking (Table 3 of [32]). Surface nicotine levels in vape-shops could even exceed nicotine levels observed in cigarette smokers’ homes and cars.”*

It is true that the density levels they found in the implanted samples are way above those measured in smokers’ homes (which peak around ~ 200 µg/m^2^, see [34,35,36]). As mentioned before, these measurements are artificial. A better comparison could be made with fixed surfaces, as in Table 1 (223.6 ± 313.4 µg/m^2^, which is still larger than in most smokers’ homes).

Son et al. are comparing apples with oranges. They compared nicotine and TSNA densities in five businesses they sampled, where anonymous vapers vaped every day while shopping, with densities in homes or hotel rooms where only one family member or hotel guest smoked. In contrast, despite the small number of family members smoking in private homes, field studies still show significant measurable amounts of surface nicotine [34,35,36].

Evidently, the amount of surface nicotine in a family unit is expected to be much less than in a business that allows many clients to vape. There is no smoking analogue of a vape shop (meaning a business in an indoor venue where smokers enter and light cigarettes all the time). The right comparison with smokers in THS studies would have to involve vapers vaping at home or in their car, without artificially implanted fabrics.

### 5.4. Vaping Analogues of THS

In the second paragraph of their introduction, Son et al. write the following:

◦ “*Exhaled e-cigarette aerosol could also deposit to indoor surfaces, leading to “thirdhand” e-cigarette aerosol (THA) exposure. Similar to thirdhand smoke exposure, THA exposure includes not only contacting residual e-cigarette aerosols on indoor surfaces, but also pathways such as aerosolization and/or evaporation and conversion to secondary toxic chemicals*”. [11].

This text reads as if Son et al. manifest with full confidence the existence of a “THA” (third-hand e-cigarette aerosol) analogous to THS, even assigning it very specific properties. However, their results do not provide any evidence of “*aerosolization and/or evaporation and conversion to secondary toxic chemicals*”. The reference [11] they cited (Burton et al.) concerns only THS under laboratory conditions. As mentioned before, the generation of a secondary aerosol has only been observed in laboratory experiments and not in THS field studies.

Son et al. and Kachatoorian et al. have only found a significant measurable surface density of adsorbed nicotine and TSNAs. These are clear signals of THS, but the largest of these densities was obtained artificially in external implanted adsorptive fabrics. However, most importantly, these densities were measurable only in an indoor environment such as a vape shop, with abnormally intense vaping activity, or, as discussed before, in laboratory studies that confine the aerosol to small chambers for extended periods. These are special environments unrepresentative of normal routine vaping.

### 5.5. Children Garments in Vape Shops

Son et al. and Khachatoorian et al. address children’s safety from the perspective of dermal or ingestion exposure to fabrics with adsorbed nicotine and TNSAs. Son et al. specifically selected a piece of baby’s clothing and other baby toys to be placed in the vape shops to measure adsorbed nicotine and TNSA on these objects. They highlight children’s exposure risks to their newly found “THA”

- “*Our study also demonstrates that nicotine can deposit or be adsorbed on baby’s clothes and toys, and that tobacco- specific nitrosamines can form and retain on baby’s clothes, highlighting children’s exposure to environmental e-cigarette aerosol and THA at home is of a particular concern.*”

Concern for children’s safety is evidently legitimate and commendable, but one wonders—what is the purpose of placing children’s garments in a vape shop (an adult venue) for 14 days? Under normal conditions (when researchers are not conducting observations), the presence of such garments in a vape shop is so out of the norm that they would not stay exposed to the vape shop environment for 14 days.

Rather than assessing risk in the context of an adult venue where children are very rarely present, it is much more appropriate to assess such risks (and children’s safety in general) in the indoor environments in which children live and spend most of their time (homes, schools, and nurseries). This approach was undertaken in field research on THS; for example, Nortrup et al. [37] sampled nicotine surface density in the homes of vulnerable children.

## 6. Assessment of Dermal Exposure

The literature we have summarized and commented on in this review (on THS and including the studies on vape shops) mentions concerning risks and potential harm from dermal exposure to surface densities of accumulated nicotine and TSNAs in indoor adsorption sinks. However, measured surface densities of nicotine and/or TSNAs alone cannot be used to determine risks due to dermal exposure.

Consider a cotton fabric of 1 m^2^ in a smoker’s house with high surface densities of 200 µg/m^2^ (nicotine) and 200 ng/m^2^ (NNK). These measurements do not imply that the residents are exposed to 200 µg of nicotine and 200 ng of NNK, as such exposure would require an extreme maximalist and unrealistic assumption of continued, uninterrupted dermal interaction with the fabric—manipulating, touching, or licking every squared cm for a sufficiently long period.

Under normal dermal exposure conditions, the area of contact with the fabric could be 10–15 cm^2^ (area of the adult fingertip), which, using the example mentioned above, would involve direct contact with only 200 µg × 10 × (cm/m)^2^ = 0.2 µg of nicotine (with a likewise 1/1000-order reduction for NNK). In addition, dermal manipulation involves a short contact time (typically seconds or minutes per event). Most dermal scenarios should involve quick and incidental events that can also be frequent and systematic in occupational exposure or in sports practice. More risky scenarios with longer duration events might occur when working for hours on a desk, or with toddlers crawling on floor carpets or fabrics on furniture or engaging in hand-to-mouth activity, but in all cases, the surface area involved in dermal contact is a small proportion of the full body skin area. A realistic assessment of dermal exposure is difficult because human activity patterns are very varied and complex.

### 6.1. Exposure to Nicotine

A quantitative assessment of dermal exposure requires a risk model based on specific exposure scenarios with input from chemical and biophysical information on skin absorption. For this purpose, we consider the chronic exposure assessment of the risk model of the United States Environmental Protection Agency (USEPA) [66] for the dermal route of organic compounds in water. In this section, we adapt this USEPA chronic risk model to a daily intake exposure that is appropriate for estimating non-cancer risks (the full chronic risk model is described in detail in the Appendix A).

We consider a daily dose (ADD_abs_) in units mg/(kg day), expressed as mass of contaminant (mg) per unit body weight (kg) through the following formula based on Equation 3.1 of [66] (see the full equation in the Appendix A):

(1) $${\mathrm{ADD}}_{\mathrm{abs}}=\frac{{\mathrm{DA}}_{\mathrm{event}}\times \mathrm{SA}\times \mathrm{EV}}{\mathrm{BW}}$$

where DA_event_ is the absorbed dose (mg/cm^2^) per event, SA is the skin area available for contact (cm^2^), EV is the event frequency (N events per day), and BW is average body weight (70 kg for adults 10 kg for toddlers). The physiological input of the absorbed substance (nicotine) is conveyed through the absorbed dose DA_event_, the formula of which is given and discussed in detail in the Appendix A.

We need to set up exposure scenarios with parameters for nicotine to evaluate Equation (1) and compare it with an appropriate non-cancer toxicological marker. We examine two simple scenarios of dermal exposure:

- Scenario 1: Short-duration dermal hand contact with samples by adults. We assume 200 events/day, with t_event_ = 10 s and SA = 15 cm^2^. The latter value is justified by the combined area of the thumb, index, and middle fingers comprising roughly 1% of the average body surface area of 1.7 m^2^ [67].
- Scenario 2: Longer duration dermal contact by toddlers (1–2 years old). Chapter 16 of USEPA Exposure Factors Handbook [68] provides observational evidence that toddlers spend on average 771 min/d = 12.85 h/d in their bedroom (Table 16—15 of [68]) and engage in 20 events of hand-to-mouth activity per hour (Table 4.1 of [66]), each lasting 30 s. Hence, we consider 240 events. For the skin area exposed we assume also SA = 15 cm^2^ considering that this dermal manipulation might involve a larger hand surface, even if toddlers’ hands are much smaller than adults’ hands.

The absorbed dose DA_event_ for nicotine is computed from Equations (A1)–(A3) in the Appendix A and then used to evaluate ADD_abs_ (1) for the two described scenarios. The resulting ADD_abs_ must be compared with an appropriate toxicological marker.

An occupational Threshold Limit Value (TLV) is given by the No Observed Adverse Effect Level (NOAEL) of the National Institute of Occupational Safety and Health (NIOSH) of 0.5 mg/m^3^ for an 8-h work shift [69], which, assuming 20 m^3^ of daily breathed air and a BW of 70 kg, leads to a maximal nicotine daily intake of 0.047 mg/kg day. The only toxicological marker we found besides this NOAEL is the Acceptable Daily Intake (ADI) of 0.0008 mg/kg body weight per day for nicotine derived by the European Food Safety Agency (EFSA) [70]. The EFSA’s ADI is a marker of the ingestion route conceived for nicotine content in various food items (not on tobacco products or e-liquids).

The use of an ingestion Reference Dose (RfD_oral_) to extrapolate a dermal absorption RfD_abs_ is justified in Chapter 4 of [66], with Equation 4.3 of [66] providing the relation *RfD*_*abs*_ = *RfD*_*oral*_ × *ABS*_*GI*_ where ABS_GI_ is the fraction of the compound absorbed in the gastrointestinal tract. Once nicotine is consumed in either route, 50–90% is converted to cotinine and excreted by urine [71,72]; therefore, the EFSA’s ADI might be a good reference marker. However, discussing whether this threshold marker provides an appropriate reference is outside the scope of this review.

**Figure 1:**
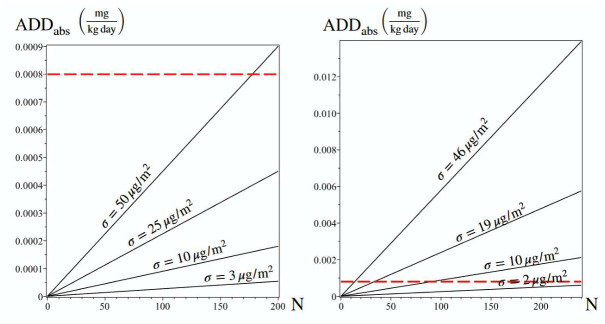
AAD_abs_ vs. N (number of events per day) for assorted nicotine surface densities s. The red line marks ADI limit (0.0008 mg/kg day), The left-hand panel corresponds to Scenario 1 and the right-hand side one to Scenario 2.

A comparison of ADD_abs_ with the ADI of EFSA is displayed in Figure 1 below. For scenario 1, the left panel shows ADD_abs_ as a function of the number of events N for various surface densities of adsorbed nicotine reported in the literature [34,35,36]. The figure shows that with up to 200 events per day, ADD_abs_ is below the ADI value of 0.0008 mg/(kg day) for all but the highest nicotine surface density of 50 µg/m^2^. For all surface densities, the curves of ADD_abs_ are two orders of magnitude below the occupational NOEL.

For scenario 2, we used nicotine surface densities in homes whose occupants were medically fragile children, reported in [36]. Since t_event_ = 30 s represents a longer duration event for hand-to-mouth activity, in this case, ADD_abs_ is significantly larger than in Scenario 1 (even with lower nicotine surface densities). The right-hand side panel of Figure 1 shows ADD_abs_ above the ADI value 0.0008 mg/(kg day) for all but the lowest nicotine surface density of 2 µg/m^2^, a density that was measured in a home in which indoor smoking was banned.

Although these two scenarios represent simple idealizations of an exposure characterized by highly variable and complex activity pattern, they do illustrate the strong dependence of daily intake on body surface area and the duration of dermal contact. The examples also show how high surface densities *σ* of adsorbed nicotine in smokers’ homes might result in non-trivial risks of dermal exposure—not so much for adults (Scenario 1) whose dermal interaction is very brief, but for toddlers engaged in frequent longer duration events (crawling and hand-to-mouth activity). Similar levels of nicotine surface density were measured in vape shops, but these venues are not representative of vapers’ homes. As we have shown in this review, surface densities of adsorbed nicotine from vaping (especially with nicotine salts) are likely to be at most comparable with the lowest concentrations displayed in Figure 1.

### 6.2. Exposure to TSNAs

Two studies have examined direct dermal exposure to adsorbed NNK in surface sinks:

- Tang et al. [13] considered published measurements of NNK in surface sinks in the range 3.2–220 ng/m^2^. Their exposure scenario was an adult (full body surface: 2 m^2^) in continuous contact with THS-contaminated bedding materials during a full sleeping cycle of 8 h, leading to a daily NNK uptake of 2–145 ng/day, surpassing the No Significant Risk Level (NSRL) of 14 ng day set by the Office of Environmental Health Hazard Assessment (OEHHA) Prop. 65.
- Wang et al. [73] measured nicotine and concentrations of TSNAs in a 100 m^3^ furnished chamber in which regimented smoking took place. They measured the total count of TSNAs accumulated on a desk used for working or studying. They computed the Incremental Lifetime Cancer Risk (ILCR) for an exposure scenario of continuous dermal contact with this desk for eight hours. Their results show a significant gradual progression to unacceptable cancer risk levels even with modestly increasing numbers of smoked cigarettes.

The risk assessments in both studies are questionable, as they are based on unrealistic continuous direct dermal exposure scenarios. If more realistic conditions of dermal contact are considered, cancer risks due to TSNAs should significantly decrease.

Tang et al.’s scenario can be only minimally realistic for indoor dwellers in hot climates, who might sleep covered by a single bedding sheet. However, in general, bedding material is not a simple surface or fabric univocally exposed to ETS that becomes contaminated by THS. Normally, this material consists of two or more layers of fabrics, with ETS deposition and possible THS effects only taking place on the external thicker layer that covers a thinner internal fabric sheet that is in direct dermal contact with the sleeping person.

Wang et al. did not estimate the surface area of dermal contact and assumed uninterrupted contact involving the full desk surface. This leads to an evident risk overestimation, since the continuous 8-h dermal contact takes place only through a small proportion of the full body skin area: the hands and arms (assuming short sleeves).

## 7. Conclusions

Considering the available evidence, both experimental (Goniewicz and Lee [27], Marcham et al. [28] and Colby et al. [29]) and observational (Khatchatoorian et al. [30,31], Son et al. [32]), we can safely state that “third-hand vaping” has not been detected under the environmental conditions in which vaping takes place among millions of users. These conditions are mostly private homes, outdoor spaces, and indoor public spaces where vaping is allowed. Vape shops, vape fests or festivals, and even social events or celebrations where many vapers vape are all sporadically attended, and thus, are not representative of environments where vaping takes place regularly on a day-to-day basis.

Nicotine adsorption and the formation of byproducts (including TSNAs) is the only observed and measured phenomenon in vaping that is common to THS. However, non-negligible measurements have only been observed under abnormal conditions, such as laboratory experiments with artificially generated aerosols or in vape shops that are sites of localized, excessively intense vaping activity.

Moreover, even under these abnormal conditions, there is no evidence of desorption and re-emission of adsorbed nicotine and byproducts or other adsorbed compounds and their byproducts. In other words, even in laboratory experiments, there is no sign of a chemical aging process as is observed in THS. The only experiment showing re-volatilization and reemission in a vaping aerosol attempted by Colby et al. [29] is extremely contrived and artificial.

When interpreting their results, the authors of the observational studies conducted in vape shops remarked that adsorbed nicotine from vaping produced much higher surface densities than smoking, comparing their numbers with those reported in homes of smokers. However, this is comparing apples with oranges, contrasting a private home or hotel room with one family member or one guest smoking with a public venue where many anonymous vapers vape. The difference in furnishing between homes and vape shops is also important in assessing the adsorption of SVOCs.

Evidently, there is much more adsorbed nicotine in the vape shop, but the right comparison must be between private homes of vapers and smokers. In addition, the modest furnishing of vape shops creates few efficient adsorption sinks in surfaces where nicotine and its byproducts may accumulate. These are very different conditions from furnished homes and other private and public venues that contain many sinks.

It is important to emphasize that smoking and vaping markets and the habits of vapers and smokers are changing rapidly. Nowadays, especially in the US and developed countries, most public spaces have become smoke-free, which involves an enormous reduction in public spaces where ETS can produce aging residues to form significantly measurable THS. This fact should decrease public interest but not necessarily the motivation to continue researching the subject, which remains a relevant public health issue, especially in regions with high levels of exposure to ETS (Eastern Europe, Asia and Middle East).

Changes in vaping are also significant. Before, 2018 most users vaped first- and second-generation devices (cig-alikes and cartomizers) and tank devices [57], all of which relied on e-liquids with free-base (non-protonated) nicotine, which is much more volatile than salt-based (protonated) nicotine. A much larger share of vapers used third-generation tank devices that released larger aerosol clouds into the environment. In addition, vape shops were social hubs where vapers congregated and vaped.

These factors (free-base nicotine, more powerful devices, and busy vape shops) strongly favor nicotine adsorption on indoor surfaces in vape shops, and perhaps even in homes or other venues. However, markets and habits changed: since the emergence of Juul 2018, salt-based protonated nicotine has become popular, and most consumers have shifted to low-powered devices: pods and disposables and low-powered tank models [58,59,60,61]. In addition, vape shops are no longer social hubs of a vaping subculture where vapers congregate. New contemporary conditions do not favor nicotine adsorption: most vapers currently exhale small quantities of non-volatile nicotine whose capacity to adsorb is uncertain.

It is important to assess dermal and ingestion exposure to “third-hand” phenomena under the best possible approximation to normal, realistic environmental conditions. Most of the literature we have reviewed (THS studies and the studies in vape shops) warn about risks from high surface densities of accumulated adsorbed nicotine and TSNAs. However, these densities do not determine actual risks for dermal exposure, which mostly involves contact with surfaces and fabrics over short time intervals and small surface areas.

Finally, in Section 6, we applied a risk model to two simplified (but minimally realistic) scenarios, showing that quantification of risks from dermal exposure to adsorbed nicotine in indoor surfaces is very sensitive to exposure conditions (time and surface of dermal contact). We show that risks from short-duration dermal contact are low (even in smoking environments) but might be concerning in cases with longer dermal contact; for example, in the case of toddlers living in smokers’ homes with high nicotine surface densities. Risks are low in normal vaping environments, given the much-reduced nicotine mass released to the environment and the negligible trace levels of TSNAs found in vaping aerosols.

## Figures and Tables

**Table 1:** Surface densities measured by Son et al. [32].

	Natural Surfaces Maximum	Implanted Surfaces			
		Maximum	Paper	Baby Clothing	Glass
Nicotine (µg/m^2^)	223.6 ± 313.2	2073	1097.2 ± 580.6	814.7 ± 732.7	325.4 ± 547.8
NNA (ng/m^2^)	4.78 ± 11.8	474.4	199.9 ± 195	57.8 ± 6.30	25.0
NNK (ng/m^2^)	44.8 ± 102.3	184.0	102.3 ± 69.4	75.6	11.4

## Data Availability

No new data were created or analyzed in this study. Data sharing is not applicable to this article.

## Abbreviations

The following abbreviations are used in this manuscript:

THS	Third Hand Smoke
ETS	Environmental Tobacco Smoke
EVA	Environmental Vaping Aerosol
MS	Mainstream emission
SS	Side-stream emission
POA	Primary Organic Aerosol
SOA	Secondary Organic Aerosol
TSNA	Tobacco Specific Nitrosamines
NNN	N’-nitrosonornicotine
NNK	4-(methylnitrosamino)-1-(3-pyridyl)-1-butanone

## Appendix A. USEPA Risk Model of Dermal Exposure

Since the vehicle associated with direct dermal exposure is adsorbed nicotine in surface sinks contained in thin aqueous films of a thickness of 100–300 nm [12], we considered the USEPA risk model for the dermal route of organic compounds in water discussed in Chapters 3–5 of [66]. The risk model defines an average daily dose ADD_abs_ in units mg/ (kg day), expressed as the mass of contaminant (mg) per unit body weight (kg) through the following formula (Equation 3.1 of [66])

(A1) $${\mathrm{ADD}}_{\mathrm{abs}}=\frac{{\mathrm{DA}}_{\mathrm{event}}\times \mathrm{SA}\times \mathrm{EV}\times \mathrm{EF}\times \mathrm{ED}}{\mathrm{BW}\times \mathrm{AT}}$$

where DA_event_ is the absorbed dose (mg/cm^2^) per event, SA is the skin area available for contact (cm^2^), EV is the event frequency (events per day), EF is the exposure frequency (days/year), ED is the exposure duration (years), BW is the average body weight (70 kg for adults 10 kg for toddlers), and AT is the average duration (days).

The physiological input of the absorbed substance (nicotine) is conveyed through the absorbed dose DA_event_ that can be obtained from first-order equilibrium Fick’s diffusion law from Equations (3.2) and (3.3) of [66]

(A2) $${\mathrm{DA}}_{\mathrm{event}}=2\mathrm{FA}\times K_{p}\times C_{w}\times \sqrt{\frac{6\tau_{\mathrm{event}}\times t_{\mathrm{event}}}{\pi }}\;\mathrm{for}\;t_{\mathrm{event}}\;\leq \;t^{*}$$

where t_event_ (in hours) is the duration (time) of the event, K_p_ (cm/h) is the chemical-specific permeability coefficient in water, C_w_ (mg/cm^3^) is the chemical concentration of the aqueous vehicle, and FA is its dimensionless absorbed fraction. The characteristic times of diffusion (all given in hours) are the lag time (t_event_) and the time (t*) to reach a steady state associated with diffusion over the stratum corneum (the most external membrane of the epidermic), given by equations A.4 and A.5 of [66]

(A3a) $$t^{*}=2.4\times \tau_{\mathrm{event}}\;(\mathrm{for}\;B\;=\;K_{p}\times \frac{\sqrt{\mathrm{MW}}}{2.6}<0.6),$$

(A3b) $$\tau_{\mathrm{event}}=\frac{L_{\mathrm{SC}}^{2}}{6D_{\mathrm{SC}}}\;=\;0.015\;\times \;10^{0.0056\;\times \;\mathrm{MW}},$$

where MW is molecular weight in mol/g, while L_sc_ and D_sc_ are, respectively, the thickness of the stratum corneum (cm) and the effective diffusion coefficient for chemical-specific transfer through the stratum corneum (cm^2^/hour). The concentration of the aqueous vehicle is C_w_ = m/V, where m is the nicotine mass of the sink (*m* = *σ* × *SA*, where *σ* is the surface density (in µg/m^2^) measured in field studies), while the volume is *V* = *SA* × *L*_*SC*_. Thus, we have *C*_*w*_ = *σ*/*L*_*SC*_ in units mg/m^3^.

For nicotine, MW = 162.2 g/mol, F = 1 is justified by Exhibits A-4 and A-5 of [66] for short-duration events and for the MW of nicotine. Regarding the permeability coefficient K_p_, it has been determined by multiple kinetic models. The review by Brown et al. [74] provides the most recent and comprehensive description of these models for hundreds of organic compounds in its supplementary file, with log K_p_ = −2.35 for nicotine. For these values, we obtain t_event_ = 0.121 h = 7.2 min, and thus (A2) is valid for events lasting less than t* = 0.29 h = 17.44 min.

Since nicotine is not carcinogenic, we focused only on daily intake by setting EF = ED = AT = 1 in Equation (A1), leading to equation (1) of Section 6 that compared with non-cancer toxicological markers: the NOAEL of NIOSH [69] and the ADI of EFSA [70].
